# Systemic Inflammation Response Index Predicts Thrombolytic Therapy Requirement in Intermediate-High Risk Acute Pulmonary Embolism: A Retrospective Study

**DOI:** 10.3390/jcm15114362

**Published:** 2026-06-04

**Authors:** Tuğba Çiçek, Kerim Yeşildağ

**Affiliations:** Department of Pulmonary Diseases, Necmettin Erbakan University School of Medicine, 42090 Konya, Türkiye; dr.tugbacicek@gmail.com

**Keywords:** acute pulmonary embolism, biomarkers, immunothrombosis, risk stratification, systemic inflammation response index, thrombolytic therapy

## Abstract

**Background:** Intermediate-high-risk acute pulmonary embolism (APE) presents a clinical challenge, as patients are hemodynamically stable at admission yet carry a substantial risk of deterioration requiring rescue thrombolytic therapy. This study evaluated whether admission complete blood count-derived inflammatory indices, particularly the Systemic Inflammation Response Index (SIRI), are associated with subsequent thrombolytic therapy requirement. **Methods:** In this retrospective cohort study, 134 patients with computed tomography pulmonary angiography–confirmed intermediate-high-risk APE, classified according to 2019 ESC guidelines, were grouped based on the need for rescue thrombolytic therapy (*n* = 52) versus no thrombolytic therapy (*n* = 82). Inflammatory indices were calculated from admission blood samples, and multivariable logistic regression and ROC analyses were performed. **Results:** Patients requiring thrombolysis had significantly higher SIRI, SII, hs-troponin I, and systolic pulmonary artery pressure (SPAP), and lower lymphocyte counts. In multivariable analysis, SIRI (OR = 2.08, 95% CI 1.37–3.13), SPAP (OR = 1.21, 95% CI 1.06–1.37), and troponin (OR = 1.01 per 10 ng/L increment, 95% CI 1.00–1.01) were independently associated with thrombolytic therapy requirement. **Conclusions:** SIRI, SPAP, and hs-troponin I were independently associated with thrombolytic therapy requirement in intermediate-high-risk APE. These findings are hypothesis-generating and warrant prospective validation before clinical implementation.

## 1. Introduction

Among cardiovascular diseases, APE ranks as the third leading cause of mortality, following acute myocardial infarction and stroke [1]. Recent large-scale epidemiological data confirm that PE continues to impose a substantial global burden, with incidence rates rising across diverse geographic regions and healthcare settings [2]. The clinical spectrum of APE is highly variable, ranging from asymptomatic peripheral clots to high-risk presentations resulting in acute right heart failure and cardiovascular collapse. Because approximately two-thirds of APE-related deaths occur within the early hours of the clinical event, rapid and accurate risk stratification is critical to guide immediate life-saving interventions, including timely reperfusion strategies such as systemic thrombolytic therapy [3].

Risk stratification in APE was based on the 2019 European Society of Cardiology (ESC) guidelines, which classify patients into four risk groups (high, intermediate-high, intermediate-low, and low risk) according to hemodynamic status, right ventricular dysfunction, and cardiac biomarkers [4]. Since this study was conducted retrospectively, the 2019 ESC classification represented the guideline framework routinely used during the study period and was therefore adopted for patient selection and risk categorization. We acknowledge that the recently published 2026 AHA/ACC/Multisociety guidelines have introduced an updated five-tier classification system integrating clinical severity, biomarkers, imaging findings, and hemodynamic parameters [5]. Nevertheless, the evolving landscape of PE risk stratification should be considered when interpreting the present findings. Within this framework, the intermediate-high-risk category poses a particularly difficult management challenge. These patients are hemodynamically stable at presentation yet exhibit concurrent right ventricular (RV) dysfunction and myocardial injury—a combination that places them at significant risk of clinical deterioration [6]. While many stabilize with standard anticoagulation, a subset will progress to hemodynamic compromise requiring rescue thrombolytic therapy [7]. Identifying this subgroup at the time of initial presentation, before deterioration occurs, remains an unmet clinical need.

The pathophysiological basis of venous thromboembolism is increasingly understood through the concept of immunothrombosis, in which the systemic inflammatory response and the coagulation cascade are inextricably linked [8]. Inflammation within the vessel wall and systemic circulation facilitates thrombus propagation through concurrent activation of immune and coagulation systems, while physiological stress and sympathetic activation lead to a rise in pro-coagulatory factors from platelets and leukocytes alongside a decline in lymphocyte counts [9,10]. Hematological indices derived from routine complete blood counts (CBC), such as the Systemic Immune-Inflammation Index (SII) and the Systemic Inflammation Response Index (SIRI), have been proposed as accessible surrogates of this thrombo-inflammatory state [11]. By integrating neutrophil, lymphocyte, platelet, and monocyte counts into composite scores, these indices may reflect the overall physiological stress burden during the acute phase of APE more comprehensively than individual cell counts alone [12]. However, existing studies have been heterogeneous in their outcome definitions and patient populations, and no study has specifically evaluated whether these indices, obtained from routine admission samples, are associated with the risk of subsequent clinical deterioration and rescue thrombolytic therapy requirement in a well-defined intermediate-high-risk cohort.

Therefore, this study aimed to evaluate whether admission SII and SIRI levels are associated with subsequent thrombolytic therapy requirement in intermediate-high-risk APE patients. We hypothesized that these indices may be associated with subsequent clinical deterioration and thrombolytic therapy requirement in intermediate-high-risk APE patients.

## 2. Results

A total of 573 patients with CTPA-confirmed APE were screened. Of these, 151 were classified as intermediate-high-risk according to the 2019 ESC guidelines. Seventeen patients were excluded according to the predefined exclusion criteria, including active infection or sepsis (*n* = 5), active malignancy or ongoing chemo/radiotherapy (*n* = 3), current immunosuppressive therapy (*n* = 3), chronic kidney failure requiring dialysis (*n* = 2), and missing or incomplete data (*n* = 4), leaving 134 patients for the final analysis. Of these, 52 patients (38.81%) subsequently developed hemodynamic deterioration and required systemic thrombolytic therapy (thrombolytic therapy group), while 82 patients (61.19%) maintained hemodynamic stability throughout hospitalization (no thrombolytic therapy group). The mean age was 64.72 ± 15.62 years; 47% were men, and 45 patients (33.58%) had deep venous thrombosis at admission.

Baseline characteristics of the study population are presented in Table 1. Patients in the thrombolytic therapy group had significantly higher monocyte counts (*p* < 0.001). Serum levels of D-dimer (*p* < 0.001), CRP (*p* < 0.001), and hs-troponin I (*p* < 0.001) were also significantly higher in the thrombolytic therapy group. Furthermore, NLR (*p* = 0.026), SII (*p* = 0.013), SIRI (*p* < 0.001), and SPAP (*p* < 0.001) values were markedly elevated. In contrast, lymphocyte counts were significantly lower in the thrombolytic therapy group (*p* = 0.014). Although SII was significantly higher in the thrombolytic therapy group on group comparison, it did not reach significance in univariate logistic regression analysis (*p* = 0.052) and was therefore not entered into the multivariable model. In-hospital mortality was significantly higher in the thrombolytic therapy group than in the no thrombolytic therapy group (9.62% vs. 1.22%, *p* = 0.04).

The results of univariate and multivariable logistic regression analyses are summarized in Table 2. In univariate analysis, monocyte count (OR = 2.12, 95% CI 1.64–2.75; *p* < 0.001), lymphocyte count (OR = 0.59, 95% CI 0.37–0.93; *p* = 0.022), D-dimer (OR = 1.12, 95% CI 1.05–1.21; *p* = 0.001), CRP (OR = 1.04, 95% CI 1.02–1.05; *p* < 0.001), troponin (OR = 1.01 per 10 ng/L increment, 95% CI 1.01–1.02; *p* < 0.001), SIRI (OR = 1.74, 95% CI 1.39–2.18; *p* < 0.001), and SPAP (OR = 1.28, 95% CI 1.16–1.40; *p* < 0.001) were significantly associated with thrombolytic therapy requirement.

In multivariable logistic regression analysis, SIRI (OR = 2.08, 95% CI 1.37–3.13; *p* = 0.001), SPAP (OR = 1.21, 95% CI 1.06–1.37; *p* = 0.005), and troponin (OR = 1.01 per 10 ng/L increment, 95% CI 1.00–1.01; *p* < 0.001) were independently associated with thrombolytic therapy requirement. D-dimer did not demonstrate independent significance after adjustment (OR = 1.05, 95% CI 0.94–1.17; *p* = 0.360).

**Figure 1:**
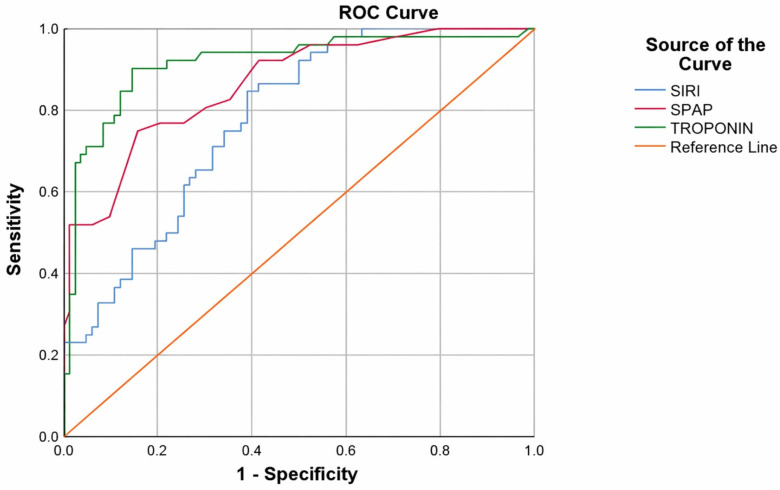
ROC curves demonstrating the discriminative ability of the three evaluated parameters.

ROC analysis demonstrated good discriminative ability for all three parameters (Figure 1). SIRI, at a cut-off value of 3.10, demonstrated discriminative ability for identifying patients who subsequently required thrombolytic therapy, with a sensitivity of 69.2% and a specificity of 68.3% (AUC: 0.78, 95% CI: 0.70–0.85; *p* < 0.001). Troponin, with a cut-off value of 50.15 ng/L, showed a sensitivity of 84.6% and a specificity of 85.4% (AUC: 0.92, 95% CI: 0.86–0.97; *p* < 0.001). SPAP, with a cut-off value of 43.50 mmHg, yielded a sensitivity of 76.9% and a specificity of 79.3% (AUC: 0.86, 95% CI: 0.80–0.93; *p* < 0.001).

## 3. Discussion

In this study of 134 patients with intermediate-high-risk APE, we identified SIRI, SPAP, and hs-troponin I as independent predictors of hemodynamic deterioration requiring rescue thrombolytic therapy. While various inflammatory markers reflected a systemic thrombo-inflammatory state in our cohort, these three parameters emerged as the most robust indicators of clinical outcome. Our findings suggest that SIRI, alongside established hemodynamic and cardiac injury markers, is independently associated with subsequent thrombolytic therapy requirement, identifying a potential adjunctive marker of thrombo-inflammatory burden in this patient population. In our cohort, the significantly higher in-hospital mortality rate observed in patients requiring rescue thrombolytic therapy (9.62% vs. 1.22%, *p* = 0.04) underscores the critical clinical risk inherent in intermediate-high-risk acute pulmonary embolism. This marked disparity in survival emphasizes the urgent need for predictive tools that can identify patients at risk of rapid hemodynamic collapse before it occurs. Indeed, numerous studies have investigated various biomarkers and clinical parameters to predict clinical deterioration in PE patients, including inflammatory markers, clinical phenotypes, and hematological indices [13,14,15]. Within this established framework, inflammatory indices derived from routine hematological parameters have been suggested to represent practical and cost-effective indicators of the systemic thrombo-inflammatory state. Building upon this body of evidence, our study specifically targeted intermediate-high-risk patients to assess the utility of these hematological parameters in predicting clinical deterioration within this high-risk subgroup.

During the course of APE, thrombotic occlusion of the pulmonary arterial bed significantly increases pulmonary vascular resistance and RV afterload. This hemodynamic strain leads to the elevation of SPAP and subsequent RV dilation and dysfunction. Existing literature indicates that SPAP is a valuable marker for risk stratification, as a strong correlation is often observed between SPAP levels, the severity of RV dysfunction, and in-hospital mortality [16,17]. In the intermediate-high-risk category, where hemodynamic instability has not yet manifested, elevated SPAP at admission may reflect the degree of underlying RV stress and thus signal a higher likelihood of subsequent clinical deterioration. Our findings strengthen these observations; SPAP emerged as a robust independent predictor of thrombolytic therapy requirement in this patient group, suggesting that its early assessment may help identify those at greatest risk of failing conservative management. This hemodynamic threshold appears to represent a critical point beyond which right ventricular compensation fails, as evidenced by the clinical deterioration requiring thrombolytic therapy observed in 38.81% of our cohort. Beyond pressure overload, myocardial injury further compounds the risk of deterioration. Complementing this pressure-related strain, hs-troponin I demonstrated the highest discriminative performance among the evaluated parameters (AUC: 0.92), supporting existing evidence that cardiac troponin levels are valuable markers for assessing myocardial stress and predicting clinical deterioration in intermediate-risk APE [18,19]. The independent association of both elevated SPAP and troponin with thrombolytic therapy requirement reinforces the link between mechanical obstruction and biological cardiac stress. Identifying these thresholds early is essential for recognizing patients who, despite appearing stable at admission, are at high-risk of rapid cardiovascular collapse. Although thrombolytic therapy decisions were formally based on objective hemodynamic deterioration criteria, the potential indirect influence of biomarker awareness on clinician behavior cannot be completely excluded due to the retrospective design.

Beyond hemodynamic and cardiac injury parameters, D-dimer levels were significantly elevated in the thrombolytic therapy group in univariate analysis; however, D-dimer did not retain independent predictive value in the multivariable model. Although D-dimer remains an indispensable diagnostic tool owing to its high negative predictive value for excluding APE [20], its prognostic utility in predicting clinical course and hemodynamic deterioration is less established. Several studies have demonstrated that D-dimer levels are insufficient for predicting in-hospital mortality or clinical worsening [21], and a weak or absent correlation between D-dimer levels and radiological clot burden has also been reported [22]. Our findings align with this body of evidence, suggesting that in the intermediate-high-risk APE population, coagulation markers such as D-dimer may be less reliable for anticipating early deterioration compared to markers of systemic inflammation—such as SIRI—or myocardial injury—such as hs-troponin I. Rather than reflecting the severity of the inflammatory and hemodynamic response, D-dimer primarily mirrors thrombus burden, which may not directly translate into the risk of acute decompensation in patients who are already anticoagulated and hemodynamically stable at presentation.

Furthermore, although N-terminal pro-brain natriuretic peptide (NT-proBNP) is widely recognized as a pivotal biomarker for risk stratification due to its ability to reflect RV wall tension [23], it was not included in our study due to limited routine availability in our hospital’s laboratory during the study period. This situation reflects a common clinical reality, particularly in healthcare settings where advanced biomarkers may not always be accessible. In such settings, parameters derived from routine blood tests—such as the inflammatory indices and troponin levels utilized in this study—may provide practical, easily obtainable data that assist clinicians in the assessment and management of patients.

In our study, SIRI emerged as an independent predictor of thrombolytic therapy requirement, a finding that underscores the intricate relationship between inflammatory pathways and the coagulation cascade in the pathogenesis of APE. By integrating three distinct leukocyte lineages—neutrophils, monocytes, and lymphocytes—SIRI serves as a unified composite parameter reflecting both acute inflammatory activation and immune regulation. Consequently, compared to individual leukocyte counts or other composite indices, SIRI may offer a more comprehensive representation of the systemic inflammatory environment associated with thrombus formation [12,24]. In our analysis, a SIRI cut-off value of 3.10 demonstrated clinically relevant discriminative ability for identifying patients at risk of hemodynamic deterioration (AUC: 0.78, 95% CI: 0.70–0.85).

It is also noteworthy that although SII levels were significantly higher in the thrombolytic therapy group, SII did not retain independent significance in multivariable analysis, whereas SIRI did. This may suggest that the monocyte component incorporated in SIRI captures an additional dimension of the thrombo–inflammatory response not reflected by SII alone [25,26]. From a physiological perspective, monocytes are recognized as critical mediators of immunothrombosis, serving as a primary source of tissue factor that initiates the extrinsic coagulation pathway and promotes fibrin deposition within the pulmonary vasculature [27,28]. By capturing this specific monocyte-driven inflammatory activity, SIRI may reflect the underlying thrombo-inflammatory burden in intermediate-high-risk APE. In our cohort, elevated SIRI was independently associated with subsequent thrombolytic therapy requirement, though prospective studies are needed to determine whether this association translates into actionable clinical utility. These findings are broadly consistent with those reported by Wang et al. [29], who demonstrated that SIRI and SII were independently associated with the diagnosis of APE and correlated with higher risk stratification categories. However, notable methodological differences exist between the two studies. Wang et al. [29] compared APE patients with a dyspneic control group without APE, and performed risk stratification by contrasting non-low-risk patients against the low-risk group—an approach that encompasses a heterogeneous patient spectrum across multiple risk categories. In contrast, our study focused exclusively on the intermediate-high-risk category, which represents the most clinically challenging subgroup where the decision to escalate therapy is neither straightforward nor uniform. By isolating this specific population and using thrombolytic therapy requirement as the outcome, our findings address a more targeted and clinically actionable question: not whether inflammation accompanies APE, but whether admission SIRI can help identify which hemodynamically stable patients are at risk of subsequent deterioration requiring rescue therapy.

From a practical standpoint, the parameters identified in this study—particularly SIRI—carry meaningful implications for real-world clinical settings. As a composite index derived entirely from routine complete blood count, SIRI requires no additional testing, specialized equipment, or increased cost, and is readily obtainable at the time of initial presentation in virtually any clinical environment. This accessibility is especially relevant in resource-limited settings or centers without immediate access to advanced imaging or specialized biomarker assays, where early identification of patients at risk of hemodynamic deterioration remains particularly challenging. In such contexts, an elevated admission SIRI may serve as an adjunctive signal of heightened thrombo-inflammatory activity, warranting closer clinical monitoring. However, it must be emphasized that the present study demonstrates association rather than incremental predictive utility, as formal reclassification analyses were not performed. SIRI should therefore not be regarded as a standalone or additive decision-making tool at this stage. These findings are best interpreted as hypothesis-generating, providing a rationale for prospective evaluation of SIRI within structured multiparametric risk assessment frameworks.

This study has several limitations. First, its retrospective, single-center design limits generalizability and may introduce selection bias; unmeasured confounding factors, such as center-specific clinical practices, could have influenced therapeutic decisions. These constraints limit the external validity of the findings, and the generalizability of the identified cut-off values—particularly for SIRI—to other populations, healthcare settings, and geographic regions cannot be assumed without independent validation. Furthermore, the primary endpoint—requirement for rescue thrombolytic therapy—while triggered by objective hemodynamic deterioration criteria defined in accordance with ESC guidelines, remains inherently dependent on real-world clinical decision-making. Future prospective studies should therefore consider pre-specified composite endpoints—such as all-cause in-hospital mortality, cardiac arrest, or escalation to mechanical circulatory support—to further strengthen the objectivity of outcome assessment. Second, the absence of NT-proBNP precluded a direct comparison with SIRI for assessing right ventricular strain, highlighting the need for future studies to determine the incremental prognostic value of SIRI over established natriuretic peptides. Finally, given our sample size, these findings should be considered hypothesis-generating; therefore, validation in larger, prospective, multicenter cohorts is warranted.

## 4. Materials and Methods

This retrospective observational cohort study included consecutive adult patients diagnosed with APE. Patients were identified through the hospital electronic medical record system, and all diagnoses had been established at the time of presentation by CTPA.

Patients were included if they were aged ≥18 years, had CTPA-confirmed APE, and had a CBC available at admission to enable calculation of inflammatory indices.

Patients were excluded if they had conditions likely to substantially affect blood cell counts or inflammatory indices at presentation, including active infection or sepsis (e.g., pneumonia, urinary tract infection, COVID-19), active malignancy and/or ongoing chemotherapy or radiotherapy, chronic inflammatory or autoimmune disease in an active phase, current immunosuppressive therapy (e.g., systemic corticosteroids, methotrexate, biologics), hematologic disorders affecting leukocyte or platelet counts, chronic kidney failure requiring dialysis or severe liver disease, and missing or incomplete key data (e.g., inability to classify APE severity or missing CBC components).

**Figure 2:**
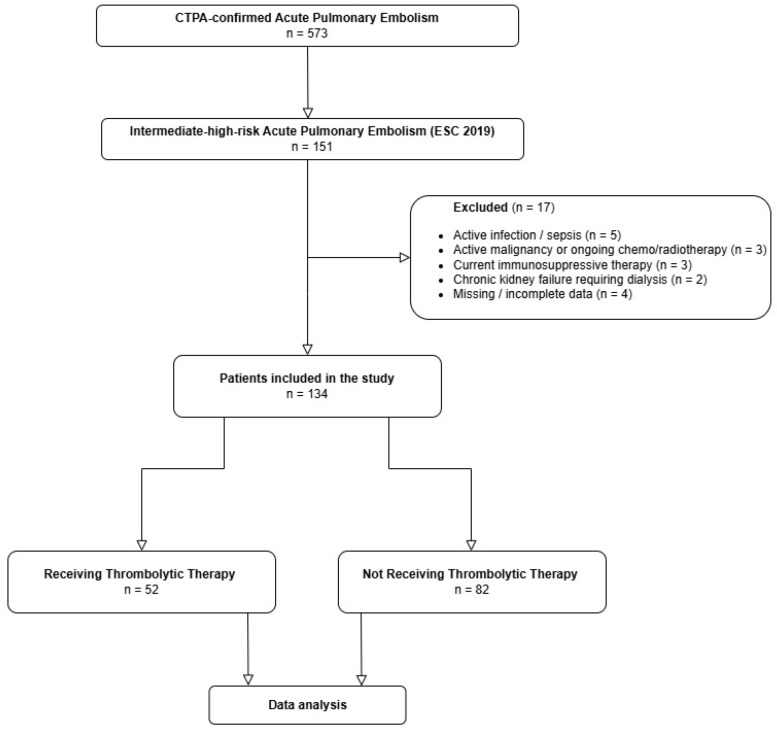
Study flow diagram illustrating patient screening and reasons for exclusion. The diagram details the number of patients assessed for eligibility, excluded, and ultimately included in the study analysis.

The primary outcome was the requirement for systemic thrombolytic therapy during hospitalization, defined as clinical deterioration with hemodynamic instability in a patient initially classified as intermediate-high-risk. The decision to administer thrombolytic therapy was made by the treating physician based on clinical assessment and in accordance with current ESC guidelines. The study flow diagram illustrating patient screening and exclusions is shown in Figure 2.

### 4.1. Definitions and Risk Classification

Pulmonary embolism was classified according to the 2019 ESC guidelines as low, intermediate-low, intermediate-high, and high-risk [4]. Intermediate-high-risk PE was defined as the absence of hemodynamic instability at presentation in the presence of both RV dysfunction and elevated cardiac biomarkers. RV dysfunction was defined as an RV/LV diameter ratio >1.0 on computed tomography pulmonary angiography or RV dilation with pressure overload on echocardiography. Elevated cardiac biomarkers were defined as hs-TroponinI levels exceeding the institutional upper reference limit of 18.5 ng/L. Although hemodynamically stable at presentation, these patients carry a substantial risk of clinical deterioration and may require escalation of therapy, including systemic thrombolysis. High-risk PE was defined by hemodynamic instability at presentation, including systolic blood pressure (SBP) < 90 mmHg or a sustained SBP decrease ≥40 mmHg for ≥15 min not attributable to new-onset arrhythmia, hypovolemia, or sepsis. Additional criteria included clinical shock with signs of end-organ hypoperfusion (e.g., altered mental status, oliguria, cool clammy extremities) or cardiac arrest requiring cardiopulmonary resuscitation. Rescue systemic thrombolytic therapy was administered exclusively to patients who, after initial classification as intermediate-high-risk, subsequently developed hemodynamic deterioration fulfilling the above-defined high-risk criteria during hospitalization. The decision to initiate thrombolytic therapy was therefore based on objective hemodynamic parameters rather than on biomarker levels, which had served solely as inclusion criteria for intermediate-high-risk classification at admission.

### 4.2. Laboratory Measurements and Index Calculations

Peripheral venous blood samples were obtained at the time of admission prior to any pharmacological intervention, including anticoagulation and thrombolytic therapy, and were analyzed using standardized automated analyzers. All indices were calculated from the first available CBC obtained at presentation. The inflammatory indices were calculated using CBC differential counts as follows:

Systemic Immune-Inflammation Index (SII) = (platelet count × neutrophil count)/lymphocyte count

Systemic Inflammation Response Index (SIRI) = (neutrophil count × monocyte count)/lymphocyte count

### 4.3. Statistical Analysis

Statistical analyses were performed using IBM SPSS 25.0 software (IBM Corporation, Armonk, NY, USA). Continuous variables were assessed for normality using the Shapiro–Wilk test and visual inspection of histograms and Q-Q plots. Normally distributed variables were presented as mean ± standard deviation, and non-normally distributed variables as median (interquartile range). Categorical variables were presented as counts and percentages. Between-group comparisons were performed using Student’s t-test for normally distributed continuous variables, the Mann–Whitney U test for non-normally distributed continuous variables, and the Chi-square or Fisher’s exact test for categorical variables, as appropriate.

The discriminative performance of inflammatory indices for identifying thrombolytic therapy requirement was evaluated using receiver operating characteristic (ROC) curve analysis. The area under the curve (AUC) with 95% confidence intervals was reported. Optimal cut-off values were determined using the Youden index, and corresponding sensitivity and specificity were calculated.

To identify variables independently associated with thrombolytic therapy requirement, binary logistic regression analysis was conducted using the enter method. Variables demonstrating univariate association at *p* < 0.05 were considered as candidate predictors. Although CRP demonstrated a univariate association with thrombolytic therapy requirement, it was not included in the final multivariable model for several reasons. First, CRP is a downstream hepatic acute-phase reactant and reflects systemic inflammatory activation rather than the cellular immunothrombotic mechanisms directly involved in APE pathophysiology. Second, the primary aim of this study was to evaluate CBC-derived inflammatory indices as practical bedside biomarkers; unlike SIRI, CRP requires a separate biochemical assay and falls outside the scope of CBC-based composite indices. Third, although both CRP and SIRI share the limitation of non-specificity, SIRI integrates neutrophil, monocyte, and lymphocyte compartments that are directly implicated in thrombo-inflammatory and immunoregulatory processes. Therefore, SIRI was retained as the primary inflammatory parameter of interest based on the predefined biological and methodological framework of the study rather than on statistical considerations alone. Although monocyte and lymphocyte counts are mathematical components of SIRI, all three variables were assessed in univariate analysis; however, monocyte and lymphocyte were not entered into the multivariable model as SIRI was retained as the primary composite inflammatory index of interest, integrating the contributions of these individual cell populations into a single composite measure. To improve clinical interpretability, troponin was rescaled to a per 10 ng/mL increment prior to multivariable analysis; odds ratios are reported accordingly. Variables that did not retain independent significance in the multivariable model were considered to lack independent association with the outcome after adjustment for other covariates.

Collinearity among candidate variables was assessed using variance inflation factors (VIF) prior to multivariable modeling. All variables included in the final model had VIF values below 5, indicating no substantial multicollinearity. The VIF values for the four variables retained in the final model were as follows: SIRI, VIF = 1.13; SPAP, VIF = 1.20; troponin (per 10 ng/L), VIF = 1.24; d-dimer, VIF = 1.10. Model calibration was assessed using the Hosmer–Lemeshow goodness-of-fit test. The final model demonstrated adequate calibration (χ^2^ = 9.518, df = 8, *p* = 0.301), indicating no significant discrepancy between observed and predicted probabilities. Results were reported as odds ratios (ORs) with 95% confidence intervals. A two-sided *p*-value < 0.05 was considered statistically significant.

No formal sample size calculation was performed, as this study was designed as a retrospective exploratory analysis aimed at generating hypotheses regarding the association between admission inflammatory indices and thrombolytic therapy requirement. To assess the analytical adequacy of the available sample for multivariable modeling, the events-per-variable (EPV) criterion was applied post hoc. With 52 outcome events and four variables entered into the multivariable model, the EPV was approximately 13, which exceeds the commonly recommended minimum threshold of 10, supporting the stability of the multivariable estimates. The findings should therefore be interpreted as preliminary, and the reported models are subject to the inherent limitations of exploratory multivariable analyses in moderate-sized cohorts.

## 5. Conclusions

In conclusion, admission SIRI, together with SPAP and hs-troponin I, was independently associated with subsequent thrombolytic therapy requirement in patients with intermediate-high-risk acute pulmonary embolism. As a parameter derived from routine complete blood count, SIRI represents a readily available and cost-effective marker of thrombo-inflammatory burden. These findings suggest that elevated SIRI at admission may reflect early biological signals of impending clinical deterioration in patients who are initially hemodynamically stable. When considered alongside established hemodynamic and cardiac injury markers, elevated admission SIRI may serve as an adjunctive signal of thrombo-inflammatory burden. However, whether SIRI provides incremental predictive value over existing tools remains to be established, as formal reclassification analyses were beyond the scope of this retrospective exploratory study. Prospective multicenter studies are therefore required to validate these findings and clarify the role of SIRI in clinical decision-making algorithms.

## Figures and Tables

**Table 1:** Baseline characteristics of the study population according to thrombolytic therapy requirement.

Variable	Study Population (*n* = 134)	Thrombolytic Therapy Group (*n* = 52)	No Thrombolytic Therapy Group (*n* = 82)	*p*-Value
Age (years)	64.72 ± 15.62	64.08 ± 13.28	65.12 ± 17.00	0.692
Male gender	63 (47.01)	23 (44.23)	40 (48.78)	0.736
Hypertension	48 (35.82)	16 (30.77)	32 (39.02)	0.432
Diabetes mellitus	34 (25.37)	16 (30.77)	18 (21.95)	0.348
Coronary artery disease	18 (13.43)	5 (9.61)	13 (15.85)	0.440
COPD	17 (12.68)	5 (9.61)	12 (14.63)	0.559
Deep vein thrombosis	45 (33.58)	23 (44.23)	22 (26.83)	0.059
WBC (×10^9^/L)	8.85 (7.27–10.95)	8.95 (7.18–11.83)	8.51 (7.27–10.69)	0.571
Hemoglobin (g/dL)	12.71 ± 1.84	12.63 ± 1.98	12.76 ± 1.75	0.711
Platelet (×10^9^/L)	225.00 (178.75–310.50)	236.00 (188.75–346.75)	216.00 (172.75–284.50)	0.130
Neutrophil (×10^9^/L)	6.61 (4.82–8.59)	6.97 (4.78–9.07)	6.43 (4.81–7.91)	0.295
Lymphocyte (×10^9^/L)	1.54 (1.10–2.18)	1.39 (1.04–1.81)	1.82 (1.16–2.36)	0.014
Monocyte (×10^9^/L)	0.68 ± 0.24	0.85 ± 0.16	0.56 ± 0.21	<0.001
D-dimer (µg/mL)	3.97 (1.46–7.95)	7.75 (5.00–11.02)	2.10 (0.98–4.58)	<0.001
Glucose (mg/dL)	109.50 (94.75–123.00)	109.50 (95.50–122.50)	109.50 (94.00–123.00)	0.718
Creatinine (mg/dL)	0.86 (0.67–1.00)	0.92 (0.71–1.04)	0.83 (0.63–0.98)	0.195
CRP (mg/L)	31.50 (14.65–72.78)	71.34 (29.75–82.06)	22.50 (12.00–48.57)	<0.001
Hs-Troponin I (ng/L)	39.45 (28.80–69.37)	81.13 (58.20–98.97)	31.85 (25.00–39.05)	<0.001
NLR	4.21 (2.54–6.43)	4.46 (3.49–6.86)	3.60 (2.02–6.19)	0.026
SII	971.62 (605.79–1710.68)	1173.38 (782.04–1818.11)	838.96 (540.08–1595.04)	0.013
SIRI	2.94 (1.68–4.74)	4.12 (2.80–5.71)	2.00 (1.21–3.89)	<0.001
SPAP (mmHg) In-hospital mortality	41.50 (35.75–46.00) 6 (4.48%)	48.00 (44.25–60.75) 5 (9.62%)	38.00 (35.00–43.00) 1 (1.22%)	<0.001 0.04

Data are presented as n (%), mean ± SD, or median (IQR). COPD—chronic obstructive pulmonary disease; CRP—C-reactive protein; NLR—neutrophil-to-lymphocyte ratio; SII—Systemic Immune-Inflammation Index; SIRI—Systemic Inflammation Response Index; SPAP—systolic pulmonary artery pressure; WBC—white blood cells.

**Table 2:** Univariate and multivariate logistic regression analysis of predictors of thrombolytic therapy requirement in patients with intermediate-high-risk acute pulmonary embolism.

	Univariate Analysis			Multivariate Analysis		
Variable	OR	95% CI	*p*-Value	OR	95% CI	*p*-Value
Monocyte	2.12	(1.64–2.75)	<0.001			
Lymphocyte	0.59	(0.37–0.93)	0.022			
D-dimer	1.12	(1.05–1.21)	0.001	1.05	(0.94–1.17)	0.360
CRP	1.04	(1.02–1.05)	<0.001			
Troponin (per 10 ng/L)	1.01	(1.01–1.02)	<0.001	1.01	(1.00–1.01)	<0.001
NLR	1.05	(0.96–1.16)	0.257			
SII	1.00	(1.00–1.01)	0.052			
SIRI	1.74	(1.39–2.18)	<0.001	2.08	(1.37–3.13)	0.001
SPAP	1.28	(1.16–1.40)	<0.001	1.21	(1.06–1.37)	0.005

CRP—C-reactive protein; NLR—neutrophil-to-lymphocyte ratio; SII—Systemic Immune-Inflammation Index; SIRI—Systemic Inflammation Response Index; SPAP—systolic pulmonary artery pressure. Troponin was rescaled to a per 10 ng/L increment to improve clinical interpretability.

## Data Availability

The datasets analyzed during the current study are not publicly available due to institutional data protection policies and confidentiality considerations related to patient records. For this reason, unrestricted public sharing of the dataset is not feasible. However, the data may be made available from the corresponding author upon reasonable request and subject to the necessary institutional approvals.

## Abbreviations

The following abbreviations are used in this manuscript:

APE	Acute pulmonary embolism
AUC	Area under the curve
CBC	Complete blood count
CI	Confidence interval
CRP	C-reactive protein
CTPA	Computed tomography pulmonary angiography
ESC	European Society of Cardiology
NLR	Neutrophil-to-lymphocyte ratio
NT-proBNP	N-terminal pro-brain natriuretic peptide
OR	Odds ratio
ROC	Receiver operating characteristic
RV	Right ventricular
SBP	Systolic blood pressure
SII	Systemic Immune-Inflammation Index
SIRI	Systemic Inflammation Response Index
SPAP	Systolic pulmonary artery pressure
SPSS	Statistical package for the social sciences
STROBE	Strengthening the reporting of observational studies in epidemiology
TF	Tissue factor
